# Exploring the Psychosocial and Economic Challenges of Elders in Debre Tabor Metropolitan City: A Holistic Perspective

**DOI:** 10.1155/jare/5665088

**Published:** 2025-11-28

**Authors:** Haileeyesus Abera Gelaw

**Affiliations:** Department of Psychology, Debre Tabor University, Debre Tabor, Ethiopia

**Keywords:** economic, elders, Ethiopia, psychosocial, qualitative design

## Abstract

The rapid increase in the global aging population presents significant psychosocial and economic challenges, especially in underdeveloped nations. This study examines the experiences of elderly retired government employees (65+) in Debre Tabor Metropolitan City, Ethiopia, using a qualitative hermeneutical phenomenological approach. Structured interviews with 15 participants revealed key issues affecting their well-being. Findings show severe isolation and loneliness, with many feeling abandoned by their families and society. Emotional distress is common, with some expressing a desire for death due to neglect and a perceived loss of purpose. Financial instability worsens their hardships, as rising inflation and inadequate pensions make necessities unaffordable. Social marginalization and economic struggles lead to declining mental and physical health, increasing vulnerability. Elder abuse is a major concern, with many experiencing neglect, psychological distress, and a lack of respect from younger generations. Malnutrition further impacts their quality of life. The study emphasizes the need for stronger social support, policy reforms, and economic measures to protect elderly individuals. Recommendations include strengthening intergenerational relationships, revising pension policies, improving healthcare access, and implementing social programs to enhance community integration and support.

## 1. Introduction

The global population is experiencing steady growth across all regions and nations, regardless of their level of development. However, this growth is occurring at a much faster rate in underdeveloped countries, particularly those with large youth populations. One of the most significant demographic trends observed worldwide is the remarkable increase in life expectancy, which has been largely driven by advancements in healthcare, improved living conditions, and better access to medical services. Between 2010 and 2015, life expectancy in industrialized nations averaged 78 years, whereas in underdeveloped countries, it was notably lower at 68 years [1]. Despite this disparity, overall life expectancy has risen considerably over the past decades, contributing to an aging global population.

In 2017, the number of individuals aged 60 and above reached 962 million, doubling since 1980. This rapid increase in the elderly population presents significant social, psychological, and economic challenges, particularly concerning health care systems, social security, and workforce dynamics. According to projections, by 2050, the global elderly population will surpass 2.1 billion, effectively doubling once again [2].

The global aging population is increasing at an unprecedented rate, with significant regional variations in the number of individuals aged 60 years and older. In 2017, Europe had 183 million people aged 60 and above, projected to rise to 247.2 million by 2050. In Northern America, the elderly population stood at 78.4 million in 2017 and is expected to grow to 122.8 million by mid-century. Similarly, Latin America and the Caribbean had 76 million elderly individuals in 2017, with projections indicating a dramatic increase to 198.2 million by 2050. Oceania, though having a smaller aging population, will see its numbers almost double from 6.9 million in 2017 to 13.3 million by 2050. Meanwhile, Asia is expected to experience one of the most significant demographic shifts, with its elderly population increasing from 549.2 million in 2017 to an astonishing 1.27 billion by 2050.

Africa, while currently having a relatively smaller aging population, is expected to witness the most rapid increase, growing from 68.7 million in 2017 to 225.8 million in 2050. This means that Africa's elderly population is projected to triple over the next few decades, highlighting the continent's unique challenges in adapting to an aging society [2]. Focusing on specific national demographics, Ethiopia provides an example of a country experiencing population growth and demographic transition. According to the Federal Democratic Republic of Ethiopia Population Census Commission [3]. Ethiopia had a total population of 73,918,505 people. Of this population, only 3.2% were aged 65 years or older.

In today's society, elderly individuals often face social alienation and marginalization. They are frequently questioned, both openly and subtly, about their abilities, contributions, and relevance, leading to a decline in their perceived self-worth. Many older adults experience limited access to resources, hold a lower social status, and have minimal influence over decisions that affect their lives [4]. Beyond societal attitudes, aging presents physical and psychological challenges that affect the well-being of older individuals. Age-related health issues, such as reduced mobility, chronic illnesses, and cognitive decline, significantly impact their ability to participate in leisure activities, maintain social connections, and perform activities of daily living. As a result, many elderly individuals experience a decline in independence, leading to helplessness, loneliness, and social isolation [5].

Elder abuse is another pressing concern that affects the psychological and physical well-being of older individuals across different regions. A Swedish study has shed light on the alarming extent of elder abuse, with findings indicating that 41.74% of elderly respondents had experienced physical assault, 39.71% had suffered psychological abuse, and 31.56% had been subjected to neglect and maltreatment [6]. 29.2% of participants felt emotional loneliness, 26.7% reported social loneliness, and 13.6% expressed both social and emotional loneliness at the same time, according to Fierloos et al. [7]. In the same spirit, Pageau et al. [8] discovered that the majority of interviewees felt lonely because they had few deep connections with other inmates. It was difficult for individuals to form friendships since they could not locate people who shared their interests.

In South Africa's Mafikeng local municipality, 64.3% of elderly men and 60.3% of elderly women reported experiencing some form of abuse. While men were more likely to suffer from physical violence, women were more frequently subjected to emotional, financial, or sexual abuse [9]. Similarly, a study conducted in the Emuhaya District of Kenya found that 81.1% of elderly individuals had experienced maltreatment at least once in their lifetime. The root causes of this abuse were linked to economic hardships, physical vulnerability, cultural norms, social discrimination, and psychological distress [10]. In addition to social and psychological challenges, food insecurity remains a significant issue for elderly populations, particularly in vulnerable communities. A study conducted in Campinas, Brazil, revealed that 15.2% of older individuals faced mild food insecurity, while 6.6% experienced moderate to severe food insecurity. This lack of consistent access to nutritious food can lead to malnutrition, exacerbating existing health conditions and increasing the risk of illness [11].

Elderly individuals living in metropolitan districts are more likely to experience poor mental health due to various socioeconomic and environmental stressors. The Geriatric Depression Scale (GDS-15) respondent ratings indicate that mental well-being among older adults is closely linked to several social factors, including housing conditions, the availability of caregivers, financial stability, and the level of family support received [12]. Beyond mental health concerns, older adults often struggle with dissatisfaction regarding changes in their health, their influence over personal care decisions, the effectiveness of rehabilitation programs, and their ability to find meaningful employment. Many elderly individuals feel that their contributions to society are undervalued, and they often face challenges in maintaining financial independence and social engagement postretirement [13].

Malnutrition is another pressing concern affecting elderly populations, particularly in developing regions. A study conducted among the Yoruba people in Nigeria revealed alarming rates of nutritional vulnerability among older adults. Only 4% of women and 10% of men were found to be at no risk of malnutrition, while the vast majority faced some level of nutritional insecurity. Specifically, 39% of men and 46% of women were categorized as highly vulnerable to malnutrition, whereas 50% of men and women were moderately susceptible [14].

The well-being of elderly individuals in Ethiopia presents significant challenges, particularly in terms of nutrition, social inclusion, and mental health. A study conducted in Eastern Ethiopia revealed that 51.7% of older adults (approximately 306 individuals) were at risk of malnutrition, while 15.7% (93 individuals) were already malnourished [15]. Similarly, research conducted at the Bet Selihome Older Adults Care Center in Oromiya confirmed the deprived living conditions of elderly residents. Many elders were socially isolated, living independently without meaningful interaction with the surrounding community. Their daily lives were characterized by monotony, as they consumed the same food regularly and lacked access to necessities, further exacerbating their vulnerability [16]. Furthermore, retirees reported higher levels of despair, lower life satisfaction, and a larger propensity for risky drinking, according to Bedaso's [17] analysis of the psychological well-being of retirees compared to older persons who continued to work (*d* = 0.49, 0.39, and *φ* = 0.65, respectively). In a similar vein, Mekonnen et al. [18] found that whereas 17.20% of individuals indicated dissatisfaction with their lives, approximately 63.68% of participants were moderately content. Kisi et al. [19] examined the economic dimension of aging, finding that approximately 83.5% of elderly households were food insecure, a figure that reveals significant economic vulnerability and its likely impact on psychosocial well-being.

Beyond physical deprivation, many elderly individuals also face psychological distress caused by negative societal attitudes. A study conducted in the Sedika Kersa community found that interactions between older adults and younger generations, including children and grandchildren, were often marked by disrespect, belittlement, and emotional neglect. Many elders felt inferior and described their experiences as abusive, humiliating, and undermining. This constant sense of devaluation led to deep-seated emotional distress, with some expressing a lack of trust in finding dependable caregivers in their later years. Some even reported feeling hopeless about the future, stating that they would rather pass away than rely on others for care and support [20].

Finally, Birhane et al.'s [21] investigation of the prevalence of loneliness among older adults reported nearly 48.69% of respondents reported feeling lonely (95% CI = 45.31%–52.07%), and Mirkena et al. [25] reported that the prevalence of depression among older adults was 41.8% (95% CI = 38.5%–45.5%), indicating a substantial mental health burden in this demographic. Similarly, elders face psychosocial challenges such as loneliness, grief, neglect, low self-worth, lack of engagement, and feeling burdensome to their families [22].

Considering the multitude of challenges facing Ethiopia's elderly population, it is crucial to gain a deeper understanding of their psychosocial and economic situation. This study explored the psychosocial and economic challenges of retired government-employed workers aged 65 and above in Debre Tabor Metropolitan City. By examining their experiences, challenges, and needs, the study aims to provide valuable insights into the well-being of elderly individuals in urban settings, helping to inform policies and interventions that can enhance their quality of life. Ensuring that aging populations receive adequate social support, healthcare, and financial stability is essential for fostering a more inclusive and respectful society that values and cares for its elderly citizens.

## 2. Methods and Material

### 2.1. Study Design and Area

A qualitative study employing hermeneutical phenomenology was conducted to examine the lived experiences of elders in Debre Tabor Metropolitan City. Established in 1334, the city served as Ethiopia's capital under Emperors Tewodros II—before relocating to Magdala—and Yohannes IV. Situated in the Amhara National Regional State, South Gonder Zone, it lies 677 km from Addis Ababa and 104 km from Bahir Dar, the regional capital, at 11° 51′ North Latitude and 38° 05′ East Longitude. With a population of 125,312, Debre Tabor operates under Metropolitan City with three city administrations and nine kebeles. The town's integrated development plan was formulated in 2000.

### 2.2. Sample Size and Sampling Procedure

For participant selection, a sample size of 15 was determined based on Creswell's [23] recommendation for phenomenological research. Participants were chosen through the purposive sampling technique. Inclusion criteria required respondents to be 65 or older, living alone, in good physical and mental health, and residing in the city for at least six months. Individuals below 65, those with compromised physical or mental health, or those with residency shorter than six months were excluded.

### 2.3. Variables and Measurements

Sex of respondents was recorded as male or female, and educational status was assessed by asking participants their highest completed level of education, categorized according to the Ethiopian system: “Ten complete” (Grade 10), “Twelve complete” (Grade 12), “12 + 2” (2-year postsecondary diploma), and “12 + 3” (3-year postsecondary program, typically a bachelor's degree). Data were collected through one-on-one structured interviews conducted between January 5 and February 2024 to gain insights into the psychosocial and economic conditions of elders. The interviews included guiding questions on social life, community treatment, life after retirement, income sufficiency, and overall psychological well-being, including emotions, thoughts, and life satisfaction.

Interviews were conducted in a designated space arranged with the South Gonder Social Security Agency and in Amharic, eliminating the need for translators as the researchers were bilingual. The interview guide was pretested on three individuals not included in the study to refine unclear questions. Each session lasted 40–50 min and was digitally recorded with participants' consent, ensuring accurate and reliable data collection.

### 2.4. Data Analysis

The data collected from participants were analyzed using thematic analysis, a methodical approach to identifying, organizing, and interpreting patterns of meaning within qualitative data. This technique enables researchers to discern shared experiences and understandings by focusing on the meanings present in the dataset. The analysis followed the six-phase process outlined by Braun and Clarke [24]:

1. Familiarization: Immersing oneself in the data to become thoroughly acquainted with its **content.**
2. Coding: Systematically labeling significant features of the data relevant to the research question.
3. Generating themes: Collating codes into potential themes, gathering all data pertinent to each candidate theme.
4. Reviewing themes: Refining themes by checking them against the dataset to ensure they accurately reflect the data.
5. Defining and naming themes: Conducting a detailed analysis to clearly define each theme and assign them descriptive names.
6. Writing up: Integrating the analytic narrative and data extracts to contextualize the analysis of existing literature. Through this process, three primary themes emerged: isolation and loneliness, wishing to die, and essential needs.

## 3. Results

The study involved eight male and seven female participants, all aged between 65 and 79 years. Each was a retired government employee receiving a pension, with educational attainment varying across the group. Specifically, one had completed Grade 10, one had completed Grade 12, nine held college diplomas, and four possessed university degrees. Participants' monthly pensions ranged from 1500 to 3500 birr. Analyses considered gender, educational background, and income in relation to the elders' circumstances, with the diversity in educational profiles providing important insights into their psychosocial and economic conditions see Table 1.

**Figure 1:**
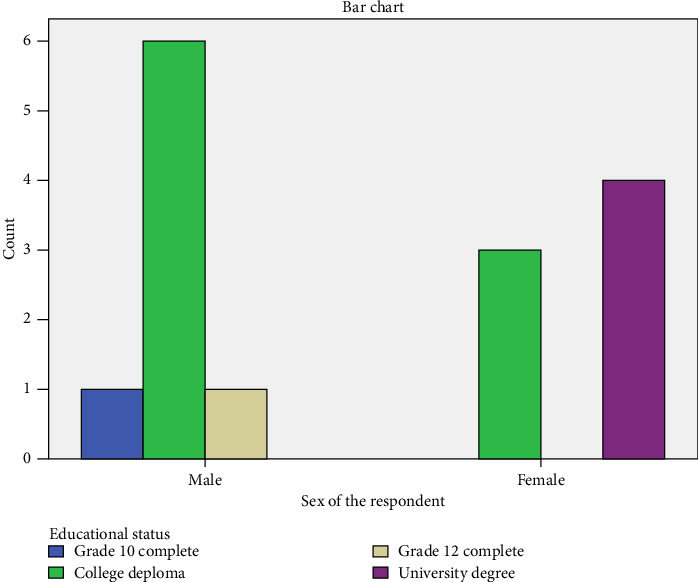
Sex of the respondent and educational status cross-tabulation.

The bar chart illustrates the relationship between the sex of respondents and their educational status Figure 1. It categorizes individuals based on their level of education and compares the distribution between males and females. Among males (*n* = 8), six individuals held a college diploma, while only one had completed Grade 10 and one had completed Grade 12, with none attaining a university degree. By contrast, the educational profile of females (*n* = 7) was concentrated at the higher end of the spectrum: four participants held university degrees, and three possessed college diplomas, with no female respondents reporting completion at Grade 10 or 12 only. This distribution suggests a gendered divergence in educational pathways, whereby males were more heavily represented at the diploma level, whereas females were more likely to have advanced to university education. The findings highlight both the prevalence of mid-level qualifications among men and the greater attainment of higher education among women within the study group, offering a useful lens for examining variations in the psychosocial and economic conditions of the elderly population (Figure 1).

**Figure 2:**
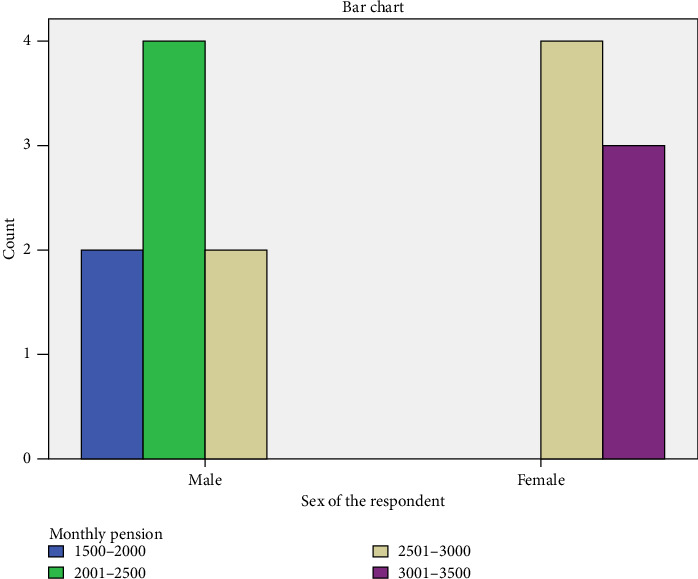
Sex of the respondents and monthly pension cross-tabulation.

The bar chart illustrates the relationship between the sex of respondents and their monthly pension distribution Figure 2 among elderly individuals. Among males, the most common pension range falls between 2001 and 2500, with four individuals receiving this amount, followed by two individuals in the 1500–2000 and 2501–3000 categories. In contrast, the majority of females receive pensions within the 2501–3000 range, with four individuals falling into this category, while three individuals receive 3001–3500, indicating a tendency for higher pensions among women compared to men. Notably, there is no female representation in the 1500–2000 category, suggesting that lower pension brackets are more common among males. This distribution highlights a potential disparity in pension earnings between genders, with females generally receiving higher pensions than their male counterparts (Figure 2).

To ensure confidentiality and maintain anonymity, unique identification codes were assigned to participants, ranging from R1 to R15. In this system, the letter “R” denotes each respondent, preventing the disclosure of personal identities while preserving the integrity of the research data.

### 3.1. Them 1: Isolation and Loneliness

A significant number of respondents expressed that, despite their dedicated efforts to contribute meaningfully to the well-being of the nation and its citizens, they have been met with neglect and disdain. They feel that society, their peers, and even the government have turned their backs on them, failing to acknowledge or appreciate the sacrifices they have made for the collective good.

We have not reflected upon any wrongdoings or sins that we may have committed, so we are left wondering: Why have we been forsaken by God? In the past, we were surrounded by a large circle of close friends, yet now, no one comes to visit us. The only time we sometimes encounter them is on the day we receive our pension. This sense of isolation is profoundly painful, especially during sacred days or when we are facing illness, as the lack of companionship during these times of vulnerability only deepens our sense of abandonment (R3, R14, and R18).

Respondents provided varying accounts of their experiences with neighbors and the broader community, highlighting the respect and acceptance they received from those around them. They spoke of being valued members of their social circles, with active participation in a variety of community events and activities. Particularly during holy days, they experienced warmth and generosity from the village residents, who would often visit them and offer thoughtful gifts as a gesture of goodwill and solidarity. These acts of kindness and connection played a significant role in reinforcing their sense of belonging and support within the community.

Having lost my family, I found myself utterly alone, with no one to turn to except for my neighbor. Their unwavering care and concern for me became evident the moment I disappeared for even a short while. They would immediately become worried, ensuring that I was safe and well. Not only did they provide emotional support, but they also took it upon themselves to look after my home and belongings, often cleaning my house and washing my clothes. I cannot help but wonder what my life would have been like if they had chosen to ignore me during those difficult times. Their kindness and compassion have been a source of immense comfort, and I truly believe that God has blessed their family and children for their selfless acts of love and support (R1, R5, R11, and R9).

### 3.2. Them 2: Wishing to Die

Twelve of the 15 participants (80%) reported experiencing a range of negative emotions, including irrational thoughts, feelings of distress, unhappiness, and a deep sense of remorse. They expressed that they often felt burdened by the societal perceptions of aging, which they believed were largely shaped by a narrow and negative view. Many felt that society tends to associate old age with disability, decline, and functional limitations, leading to a diminished sense of self-worth and purpose. This perception, they argued, perpetuates a stigma around aging, which further compounds their emotional and psychological struggles, making it even harder to navigate the challenges they face in their later years.

We have witnessed a gradual decline in our physical, emotional, and cognitive abilities, yet no one seems willing to acknowledge or comprehend the struggles we are facing internally. The changes occurring within us, both visible and invisible, go unnoticed and unaddressed, leaving us feeling increasingly invisible and insignificant. There is a deep sense of neglect, as no one seems to care about our whereabouts or well-being. This lack of attention and empathy has led us to believe that, in the eyes of others, we have become less valuable, as if our worth has diminished simply because of the inevitable aging process. This emotional isolation only exacerbates the challenges we already face. (R5, R12)

Additionally, the majority of elderly respondents expressed discomfort when reflecting on the stark contrast between their current lives and those of their younger years. Many longed for the past, yearning to recapture the vitality and experiences of their earlier years, which they regarded as some of the most thrilling, enriching, and unforgettable periods of their lives. These earlier years, filled with energy, hope, and memorable moments, seemed far removed from their present circumstances. The comparison between the past and the present often evokes a sense of longing and regret, as they wish to relive the times when life felt more fulfilling and their potential appeared boundless.

When I was with my wife and children, life felt much more fulfilling and vibrant. The time we spent together was filled with joy and meaningful experiences, and we shared many wonderful moments as a family. They had lived a happy and content life by my side, but sadly, they were no longer here. Now, my life is marked by a sense of stress and solitude, as I navigate the challenges that come with aging and loss. Despite the difficulties I face on a daily basis, I still find some solace in the fact that my situation is better than the alternative of having to survive on the streets, where I would face even greater hardship and uncertainty. Though the current reality is far from what it once was, there remains a sense of gratitude for the stability I still have. (R9)

### 3.3. Them 3: Necessities

Thirteen participants (87%) reported concerns about the rising costs of essential goods and services. They explained that these increasing expenses have become a heavy burden in their daily lives. They highlighted that the price of 5 L of oil has reached approximately 1000 birr, while 100 kg of teff, a staple food, now costs around 7000 birr. The price of 1 kg of beef has also surged to 1000 birr. Additionally, housing rents have begun to rise steadily, further contributing to the financial strain. Despite these escalating costs, the low pensions they receive are grossly insufficient to cover their basic needs. The gap between their fixed retirement income and the growing cost of living has left many struggling to make ends meet, forcing them to live in constant financial anxiety.

Three years ago, life was much simpler for me. With my modest monthly pension, I was able to cover my basic needs and live with a sense of relative stability. However, as you can observe from my current appearance, things have taken a significant turn for the worse. The strain of daily life has become more apparent, and I now find myself facing numerous hardships. While I have learned to cope with the discomfort of hunger, the most distressing challenge I now face is the inability to maintain my hygiene due to the escalating cost of necessities like detergent. This financial burden has greatly impacted my ability to care for myself, and it is a constant source of anxiety. The fact that something as fundamental as cleanliness is beyond my reach is deeply troubling and only adds to the overwhelming sense of vulnerability I now experience. (R15)

Another significant challenge faced by elderly individuals, as highlighted by the participants, is the ever-increasing inflation in the prices of essential commodities and social services. This inflationary trend has made it increasingly difficult for older persons to manage their day-to-day expenses, as the cost of basic goods and services continues to rise at a rate far beyond their limited income. As a result, many elderly individuals find themselves struggling to afford even the most basic necessities, such as food, medication, and healthcare services. The impact of rising prices on their quality of life is profound, leaving them feeling financially insecure and vulnerable. This issue of inflation not only exacerbates their economic challenges but also adds to the emotional and psychological strain they experience in their later years.

Surviving has become increasingly difficult due to the excessively high and volatile inflation that has caused the prices of essential commodities to fluctuate drastically. The soaring costs of necessities make it nearly impossible for elderly individuals to meet their daily needs, and even more troubling is the fact that seeking medical care has become a financial impossibility. When I fall ill, the expense of visiting a hospital is simply beyond my means, leaving me without the necessary healthcare I require. Moreover, I have been residing in a small, aging house that is poorly furnished, and lacking the comfort and basic amenities that one would expect. My shoes and clothes are wearing out with time, yet I have no means to replace them. These compounded challenges have significantly diminished my quality of life, and the weight of financial strain continues to grow heavier with each passing day. (R1)

## 4. Discussion and Implication

The research findings highlight critical psychosocial and economic challenges faced by the elderly, including feelings of isolation, loneliness, disrespect, and social marginalization. The study aligns with previous research conducted by Zelalem et al. [20] in Ethiopia's Sedika Kersa community, which revealed that older individuals often feel undervalued by their family members and society at large. Similarly, Cheng et al. [5] found that elderly individuals frequently experience dependency, helplessness, and social isolation. These findings collectively suggest that aging is accompanied by not only physical and economic challenges but also significant emotional and psychological burdens. Retirees reported higher levels of despair, lower life satisfaction, and a larger propensity for risky drinking, according to Bedaso [17] analysis of the psychological well-being of retirees compared to older persons who continued to work (*d* = 0.49, 0.39, and *φ* = 0.65, respectively). In a similar vein, Mekonnen et al. [18] found that whereas 17.20% of individuals indicated dissatisfaction with their lives, approximately 63.68% of participants were moderately content.

The implications of these findings are profound, as they emphasize the need for stronger familial, societal, and policy-driven support systems for the elderly. The perception of aging as a “functional constraint” contributes to stereotypes that further alienate older individuals from active social participation. This underscores the necessity of intergenerational integration, where younger generations are encouraged to maintain close relationships with older family members, acknowledging their contributions and ensuring they feel valued.

The findings of this study further emphasize the psychological distress and emotional burden experienced by elderly individuals, including feelings of remorse, regret, discomfort, pessimism, negative attitudes, and despair. These results align with previous research by Lam & Boey [12], which found that older adults in urban Hong Kong were at risk of poor mental health due to their disadvantaged social conditions. Similarly, Saveman et al. [6] highlighted the prevalence of elder abuse, with 74% of cases involving physical abuse, 71% psychological abuse, and 56% neglect or ill-treatment. Birhane and Demessie's [21] investigation of the prevalence of loneliness among older adults reported that nearly 48.69% of respondents felt lonely (95% CI = 45.31%–52.07%), and Mirkena et al. [25] reported that the prevalence of depression among older adults was 41.8% (95% CI = 38.5%–45.5%), indicating a substantial mental health burden in this demographic.

Similarly, elders face psychosocial challenges such as loneliness, grief, neglect, low self-worth, lack of engagement, and feeling burdensome to their families [22]. Moreover, 29.2% of participants felt emotional loneliness, 26.7% reported social loneliness, and 13.6% expressed both social and emotional loneliness simultaneously, according to Fierloos et al. [7]. In the same spirit, Pageau et al. [8] discovered that the majority of interviewees felt lonely because they had few deep connections with other inmates. It was difficult for individuals to form friendships since they could not locate people who shared their interests.

These alarming statistics reflect the widespread mistreatment and vulnerability of elderly populations, particularly in environments where they lack adequate social and economic support. The implications of these findings are profound, suggesting that aging is often accompanied by psychological distress, social isolation, and even abuse, leading many elderly individuals to experience deep dissatisfaction and hopelessness in their later years. The sense of worthlessness and despair they feel may stem from a lack of social integration, financial dependence, and declining health, reinforcing the need for targeted interventions.

The results of this survey reveal the severe economic hardships faced by elderly individuals, characterized by poor living conditions, inadequate nutrition, and a lack of necessities. Many elders struggle to meet their daily needs, often surviving on just one meal per day, lacking a balanced diet, proper clothing, and essential healthcare. They also reside in uncomfortable dwellings that do not meet basic living standards. These findings are consistent with Abdu et al. [15], who reported that 51.7% of elders in eastern Ethiopia were at risk of malnutrition, while 15.7% were already malnourished. Additionally, Teka et al. [16] found that elderly individuals led a self-sufficient yet deprived lifestyle, consuming the same limited diet and lacking essential amenities. Kisi et al. [19] examined the economic dimension of aging, finding that approximately 83.5% of elderly households were food insecure, a figure that reveals significant economic vulnerability and its likely impact on psychosocial well-being. Similarly, Olasunbo & Ketiku [14] highlighted the high susceptibility of elderly individuals to economic instability, with a significant proportion being moderately or highly vulnerable.

These findings underscore the severe financial strain and economic marginalization of the elderly, particularly in the face of rising inflation and inadequate pension systems. The meager monthly pension income is insufficient to cover necessities such as food, rent, clothing, and healthcare, forcing many elders into poverty and deprivation. Furthermore, the findings highlight the critical need for better healthcare access and nutrition programs to prevent malnutrition and ensure overall well-being.

## 5. Limitations

This study specifically examined the psychosocial and economic conditions of government-employed retired elders aged 65 and above who live alone in Debre Tabor metropolitan city. However, it did not include the experiences of other elderly individuals outside this demographic group. While broadening the scope to include a wider range of older persons might not have significantly altered the core findings, it would have provided a more comprehensive understanding of the psychological, social, and economic challenges faced by the elderly population. This narrow focus represents a limitation of the study, as the findings may not fully capture the diverse experiences of all older individuals, particularly those with different employment histories, living arrangements, or socioeconomic backgrounds.

## 6. Conclusion

The findings of this study highlight the severe psychosocial and economic challenges faced by elderly individuals, particularly retired government employees living alone. Many respondents experience profound isolation and loneliness, feeling abandoned by society and struggling with the psychological burden of aging. Their diminished social roles, lack of companionship, and the stigma associated with old age contribute to emotional distress and, in some cases, a desire to die. Additionally, the financial struggles faced by the elderly due to rising inflation, inadequate pensions, and limited access to necessities have exacerbated their hardships, making it difficult for them to maintain their well-being. The cumulative effects of these struggles suggest an urgent need for social, economic, and policy interventions to improve the quality of life for older individuals and ensure that they age with dignity.

### 6.1. Recommendations

1. Strengthening social support systems
  • Encourage community-based programs to foster social connections and reduce isolation among elders.
  • Promote intergenerational bonding initiatives, where younger generations actively engage with and support the elderly.
  • Establish psychosocial support services, including counseling, group therapy, and peer support networks.
2. Enhancing economic support
  • Revise pension policies to adjust for inflation and ensure that pensions adequately cover living costs.
  • Implement targeted financial assistance programs for low-income and vulnerable elderly individuals.
  • Provide subsidized essential goods and healthcare services to ease the financial burden on elderly citizens.
3. Improving access to healthcare
  • Expand free or low-cost medical services for elderly individuals, particularly those with chronic illnesses.
  • Increase government funding for home-based care and elderly-friendly health services.
  • Raise awareness about mental health issues among the elderly and integrate psychological support into healthcare services.
4. Advocating for elder rights and inclusion
  • Implement public awareness campaigns to combat age-related discrimination and stigma.
  • Encourage government and non-government organizations to prioritize elderly welfare in social policies.
  • Establish elderly-friendly infrastructure and housing programs to improve living conditions.

By addressing these challenges, policymakers and society can ensure a more inclusive and supportive environment for older individuals, enabling them to live their later years with dignity, security, and a sense of belonging.

## 7. Declaration

I declare that this research article is my original work and has not been submitted for publication elsewhere. All sources of information have been properly acknowledged.

## Figures and Tables

**Table 1:** Sociodemographic characteristics of elderly respondents by gender (*N* = 15).

Variable	Category	Female (*n* = 7)	Male (*n* = 8)
Educational status	College Diploma	3 (42.9%)	6 (75.0%)
	University Degree	4 (57.1%)	—
	Grade 10 Complete	—	1 (12.5%)
	Grade 12 Complete	—	1 (12.5%)

Monthly pension (ETB)	1500–2000	—	2 (25.0%)
	2001–2500	—	4 (50.0%)
	2501–3000	4 (57.1%)	—
	3001–3500	3 (42.9%)	2 (25.0%)

Age group (years)	65–69	3 (42.9%)	2 (25.0%)
	70–74	3 (42.9%)	3 (37.5%)
	75–79	1 (14.3%)	2 (25.0%)

## Data Availability

The data that support the findings of this study are available from the corresponding author upon request.
